# Insights into social disparities in smoking prevalence using Mosaic, a novel measure of socioeconomic status: an analysis using a large primary care dataset

**DOI:** 10.1186/1471-2458-10-755

**Published:** 2010-12-07

**Authors:** Aarohi Sharma, Sarah Lewis, Lisa Szatkowski

**Affiliations:** 1 University of Nottingham Division of Epidemiology and Public Health, Clinical Sciences Building, Nottingham City Hospital, Nottingham, NG5 1PB, UK; 2 UK Centre for Tobacco Control Studies and University of Nottingham Division of Epidemiology and Public Health, Clinical Sciences Building, Nottingham City Hospital, Nottingham, NG5 1PB, UK; 3 UK Centre for Tobacco Control Studies and University of Nottingham Division of Primary Care, Queen's Medical Centre, Nottingham, NG7 2UH, UK

## Abstract

**Background:**

There are well-established socio-economic differences in the prevalence of smoking in the UK, but conventional socio-economic measures may not capture the range and degree of these associations. We have used a commercial geodemographic profiling system, Mosaic, to explore associations with smoking prevalence in a large primary care dataset and to establish whether this tool provides new insights into socio-economic determinants of smoking.

**Methods:**

We analysed anonymised data on over 2 million patients from The Health Improvement Network (THIN) database, linked via patients' postcodes to Mosaic classifications (11 groups and 61 types) and quintiles of Townsend Index of Multiple Deprivation. Patients' current smoking status was identified using Read Codes, and logistic regression was used to explore the associations between the available measures of socioeconomic status and smoking prevalence.

**Results:**

As anticipated, smoking prevalence increased with increasing deprivation according to the Townsend Index (age and sex adjusted OR for highest vs lowest quintile 2.96, 95% CI 2.92-2.99). There were more marked differences in prevalence across Mosaic groups (OR for group G vs group A 4.41, 95% CI 4.33-4.49). Across the 61 Mosaic types, smoking prevalence varied from 8.6% to 42.7%. Mosaic types with high smoking prevalence were characterised by relative deprivation, but also more specifically by single-parent households living in public rented accommodation in areas with little community support, having no access to a car, few qualifications and high TV viewing behaviour.

**Conclusion:**

Conventional socio-economic measures may underplay social disparities in smoking prevalence. Newer classification systems, such as Mosaic, encompass a wider range of demographic, lifestyle and behaviour data, and are valuable in identifying characteristics of groups of heavy smokers which might be used to tailor cessation interventions.

## Background

Smoking remains the single greatest cause of preventable illness and premature mortality and it is estimated that the cost of treating smoking-related illness in the UK is now £2.7 billion each year[1]. Smoking is strongly linked to socio-economic disadvantage; in 2008, 27% of adults living in households in England headed by someone in a manual occupation smoked, compared to 16% in non-manual households[2]. Consequently, smoking is the largest contributor to health inequalities between the rich and the poor in the UK; it is estimated that more than half the difference in survival to 70 years of age between social classes I and V may be due to the higher smoking prevalence in class V[3]. Whilst smoking prevalence has declined over recent decades, this fall has been less marked in the more socioeconomically disadvantaged groups, so that the gap between smoking prevalence in higher and lower socioeconomic groups has widened[2]. Reducing smoking prevalence, especially in disadvantaged groups, is therefore essential to improving life expectancy, cutting health care costs, and reducing health inequalities. Identifying, measuring and attempting to explain socio-economic disparities in tobacco use are important first steps in developing strategies and targeting resources to reduce them.

It is widely recognised that traditional measures of socio-economic status have limitations and may underplay the extent of socio-economic disparities in smoking prevalence. The most frequently-used measures of socio-economic status for monitoring health in the UK are those based solely on occupation. Recent data demonstrate a higher smoking prevalence in routine and manual occupational groups (29%) than the 21% found in the population overall[2], though this figure falls well short of the prevalence of 75% or more found in studies of select disadvantaged groups[4,5]. Area-based measures, such as the Townsend Index of Deprivation[6], are often more easily ascertained than individual-level measures of deprivation, and are likely to reflect important area-level determinants of health and lifestyle. However, those in common usage combine data on a relatively small range of factors, just four census items in the case of the Townsend Score (unemployment, car ownership, housing tenure and household overcrowding), and are therefore likely to fail to capture important socio-economic determinants of smoking.

Mosaic is a UK geodemographic classification system, developed by Experian as a consumer segmentation and marketing tool[7]. Using data on over 400 variables from multiple sources, Mosaic classifies postcode areas into 61 'types' and 11 'groups' in terms of demographics, lifestyle characteristics and behaviours. Mosaic may provide a novel tool with which to identify new aspects of the socio-economic differentials in smoking behaviour in the UK. We have, therefore, used Mosaic, alongside the Townsend Index, to examine smoking prevalence within patients in a large primary care dataset, The Health Improvement Network (THIN)[8].

## Methods

### The THIN Dataset

THIN is a large dataset of electronic medical records from over 400 general practices throughout the UK[8], and contains data for approximately 6.8 million patients, over 2 million of whom are currently alive and can be followed prospectively. The dataset is broadly representative of the UK population in terms of patient age and sex, though mortality rates 5% lower than national figures suggest the dataset may slightly under-represent more deprived populations[9].

All patients over the age of 16 and registered with a THIN practice on 1st January 2008 were identified. Of these, patients who registered with a practice within the previous three months, and who were therefore less likely to have had their smoking status recorded, were excluded (the 2004 GP contract requires that smoking status of newly-registering patients is documented within three months for this recording to be financially rewarded[10]), leaving a sample of 2,426,370 individuals for analysis. These patients' medical records were searched for the last smoking-related Read Code documented in their notes before the index date, which was then used to classify patients as current, or non-current, smokers. Patients with no mention of smoking in their medical records were deemed to be non-smokers - it has been shown previously that this assumption produces smoking prevalence estimates in THIN in line with national statistics[11].

EPIC, the providers of THIN, mapped the postcode of each patient in THIN to the area's Mosaic type and group and Townsend score; the latter was provided as a categorical variable corresponding to national quintiles of deprivation in order to preserve patient anonymity.

### Mosaic

Mosaic is a tool designed to enable businesses to understand consumers' demographic and lifestyle characteristics and ensure they target their products or services at the right people, in the right locations. The tool is an area-based classification system which allocates individuals to one of the 11 Mosaic groups or 61 types based on the nature of the people living within the same postcode area. The classification is carried out at the level of the full UK postcode, equivalent to approximately 15 households, and so all individuals living in these households will be assigned to the same Mosaic category according to their 'average' characteristics[7].

Approximately one third of the variables used to classify people are derived from the UK decennial census and the remainder from a combination of public and Experian-proprietary datasets. These include property valuations, house sale prices, self-reported lifestyle surveys, a survey of adults' consumption of products, brands and media, and intelligence gathered through monitoring internet use[7]. Detailed algorithms to explain how these variables are combined to assign each UK postcode to a Mosaic group and type are not available from Experian due to commercial sensitivities.

Mosaic data were provided for each individual in THIN according to their postcode, categorised into 61 'types', and their aggregated 11 broader 'groups'. Table 1 summarises the characteristics of the 11 Mosaic groups, showing the group name assigned by Experian and a brief description of the individuals in that group.

**Table 1:** Description of the 11 Mosaic Groups[7]

Group name	Brief description
A - Symbols of success	Successful professionals of high net worth, living in fashionable areas

B - Happy families	Young families, living in newer homes, whose parents have secure positions in large organisations

C - Suburban comfort	Older, established, financially-stable families living in suburban areas

D - Ties of community	Close communities of workers in manual professions, living in inner city areas and manufacturing towns

E - Urban intelligence	Students, recent graduates, and young professionals, living in places of transient populations

F - Welfare borderline	Living in council accommodation with employment instability

G - Municipal dependency	Low income families, living in social housing

H - Blue collar enterprise	Practical, enterprising families living in homes bought from social landlords

I - Twilight subsidence	Elderly, reliant on social housing and state benefits

J - Grey perspectives	Retired but physically and financially independent

K - Rural isolation	Those living in communities in the countryside, away from urbanisation

A fuller description of each type and group was obtained from the Mosaic Interactive Guide[12], an interactive program available from Experian which provides, for each of the 11 groups and 61 types, a photo collage that gives a snapshot of the characteristics of people in that particular category, and written descriptions of their main features such as typical housing types, income, and residents' attitudes towards the area they live in. In addition, the Interactive Guide describes the distribution of categories throughout the UK and ranks categories according to their relative performance across the variety of measures used to build Mosaic.

### Analysis

Initially, the proportion of THIN patients who were current smokers was estimated by quintiles of the Townsend Index of Deprivation. Odds ratios, unadjusted and adjusted for age and sex, were obtained by logistic regression. Then, the prevalence of current smoking within each of the eleven Mosaic groups and each of the 61 types was calculated, and the magnitude and range of prevalence figures compared with those calculated previously for each Townsend quintile. Again, logistic regression was carried out to obtain odds ratios for being a current smoker in each Mosaic group and type, both unadjusted and adjusted for age and sex. All analyses were completed using STATA version 11.0 (STATA Corp, College Station, TX).

The Interactive Guide[12] was used to conduct a qualitative exploration of the common characteristics of people living in the ten Mosaic types with the highest smoking prevalence, and the ten Mosaic types with the lowest smoking prevalence, and to attempt to identify any groups with unexpectedly high or low prevalence.

Ethical approval: This study was approved by the Derbyshire Research Ethics Committee.

## Results

Of the 2,426,370 patients aged 16+ analysed in this study, 82% resided in England, 8% in Scotland, 6% in Wales and 4% in Northern Ireland, in line with official population estimates[13]. The average age of patients was 47.1 years and 49.3% were male. A Read Code documenting smoking status was available for 87.5% of patients; the proportion of patients with no record of smoking status in their notes increased from 11.3% of patients in the least deprived Townsend quintile to 13.6% in the most deprived, and ranged from 9.3% of those in Mosaic group I to 15.2% of those in group E. Overall, 20.8% of patients were recorded as current smokers in their medical records (22.5% of men and 19.1% of women).

**Figure 1:**
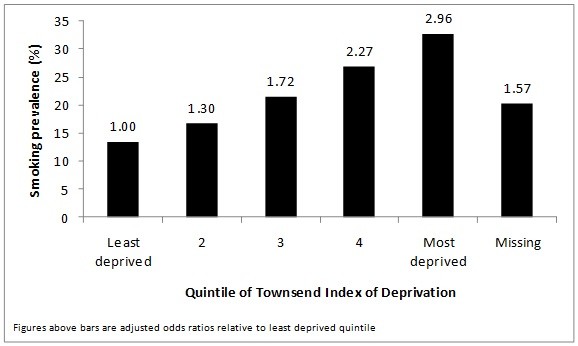
**Smoking prevalence and odds ratios by Townsend quintile**.

A Townsend score was available for 86.9% of patients. As Figure 1 shows, smoking prevalence increased across the quintiles of Townsend score, from 13.5% in the least deprived quintile to 32.7% in the most deprived quintile (p-value for test of trend <0.001). In the logistic regression analysis adjustment for age and sex made little difference to the results and, therefore, adjusted odds ratios only are presented. The odds of being a current smoker was increased almost three-fold in those in the most deprived quintile compared to the lowest quintile (adjusted OR 2.96, 95% CI 2.92 to 2.99).

**Figure 2:**
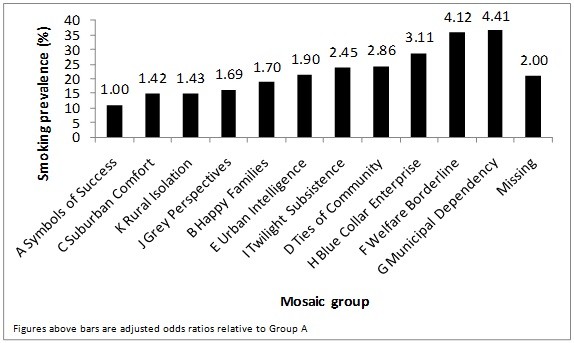
**Smoking prevalence and odds ratios by Mosaic Group**.

A Mosaic classification was available for 94.1% of patients, 7.2% more than the percentage of patients for whom a Townsend score is available. As Figure 2 (ranked with categories in order of increasing prevalence) shows, prevalence was highest in group G ("Municipal Independence") at 36.8% and lowest in group A ("symbols of Success") at 11.1%. The odds of current smoking was increased over four fold in group G compared to group A (adjusted OR 4.41, 95% CI 4.33 to 4.49).

A brief description of the ten Mosaic types with highest and lowest smoking prevalence is shown in Table 2. From the fuller descriptions available in the Mosaic guide, the 10 types with highest smoking prevalence were characterised by households that are mostly occupied by single residents, often single parents (G42, F37, F40, G41). Types D24 and H47 consist of cohabiting couples with children. The typical age of people in these Mosaic types is under 34, with the exception of types G43 (65-84 years) and F39 (65-84 years).

**Table 2:** Mosaic types with the highest and lowest smoking prevalence[12]

Mosaic Type	Description	Smoking Prevalence (%)
G41 Families on Benefits	Families, many single parent, in deprived social housing on the edge of regional centres	42.7

F40 Sharing a Staircase	Older tenements of small private flats often occupied by highly disadvantaged individuals	42.6

F38 Tower Block Living	Singles, childless couples and older people living in high rise social housing	39.6

F37 Upper Floor Families	Young families living in upper floors of social housing	39.5

G42 Low Horizons	Families with school age children, living in very large social housing estates on the outskirts of provincial cities	37.9

F39 Dignified Dependency	Older people living in crowded apartments in high density social housing	36.3

H47 New Town Materialism	Social housing, typically in 'new towns', with good job opportunities for the poorly qualified	34.4

G43 Ex-industrial Legacy	Older people, many in poor health from work in heavy industry, in low rise social housing	32.8

F35 Bedsit Beneficiaries	Young people renting hard to let social housing often in disadvantaged inner city locations	32.1

D24 Coronation Street	Low income families living in cramped Victorian terraced housing in inner-city locations	31.8
.	.	.
.	.	.
.	.	.
C15 Close to Retirement	Senior white-collar workers many on the verge of a financially secure retirement	13.1

A07 Semi-Rural Seclusion	Well-paid executives living in individually designed homes in rural environments	12.6

A06 High Technologists	Successful, high earning couples with new jobs in areas of growing high tech employment	12.0

B10 Upscale New Owners	Financially better-off families living in relatively spacious modern private estates	11.9

J51 Sepia Memories	Very elderly people, many financially secure, living in privately owned retirement flats	11.7

A05 Provincial Privilege	Senior professionals and managers living in the suburbs of major regional centres	11.1

J53 High Spending Elderly	Financially secure and physically active older people, many retired to semi-rural locations	10.8

A02 Cultural Leadership	Highly educated senior professionals, many working in the media, politics and law	10.7

A03 Corporate Chieftains	Successful managers living in very large houses in outer suburban locations	9.1

A04 Golden Empty Nesters	Financially secure couples, many close to retirement, living in sought-after suburbs	8.6

Almost all of the ten Mosaic types with the highest smoking prevalence are described as typically having few qualifications, and the majority are either unemployed or have manual occupations. The exception to this pattern is type F35, who are a diverse group comprising both disadvantaged young people as well as university graduates. A significant number of people have few, if any, qualifications, but the proportion of people with university degrees holding professional positions is well above the national average. Annual household income is below £7,499 for seven of the ten Mosaic types with the highest smoking prevalence, and below £24,999 for the other three.

The typical property types occupied by Mosaic types with high smoking prevalence are a mixture of houses and flats in urban or suburban locations, with 'public rented' ownership being most characteristic. With the exception of H47, none of the types have access to a car, and all types feel that they live in an area where there is little community support. Of the ten Mosaic types, nine are receptive to communication channelled through TV, eight to telemarketing, and seven to the tabloid press.

Of the 10 Mosaic types with lowest smoking prevalence, almost all are characterised by households that include married couples, with J51 as the exception, though individuals in this category are often widowed. The most typical age is between 45 and 64, and all types are described (with the exception of J51) as having degree level qualifications. All types not characterised as retired are within professional employment. Average annual household income for most types that are not retired is over £50,000, apart from types A04 and B10 who have a typical income between £25,000 and £49,999. The typical property type occupied by those in groups with the lowest smoking prevalence are houses owned outright, in suburban, semi-rural, or, in the case of type J51, seaside locations. All types, again except for J51, have easy access to a car, and every type feels that they live in a good area with support from neighbours. These groups are likely to be receptive to communication via broadsheet newspapers and the internet.

## Discussion and Conclusions

The Townsend Index of Material deprivation and Mosaic provide two different ways of profiling an individual's social circumstances in terms of the area in which they live. Amongst patients in the large primary care dataset of THIN, we have shown clear socioeconomic differences in smoking prevalence according to both of these measures. When using the Townsend Index, we found smoking prevalence to be progressively higher in those living in more deprived areas, in accordance with previous cross-sectional studies carried out in the UK that used the Townsend Index to indicate deprivation[14]. When using Mosaic groups, the likelihood of being a current smoker is highest in groups F and G, groups that are dependent on social benefits, compared with group A, which encompasses the most affluent members of the population.

However, the range of estimates of smoking prevalence is greater across the 11 Mosaic groups and even more so across the 61 Mosaic types compared to the difference across Townsend quintiles. For example, the highest prevalence observed in the Mosaic types was 42.7%, whilst the Townsend quintile with the highest proportion of current smokers had a prevalence of 32.7%. Smoking prevalence in the Mosaic group with the lowest proportion of current smokers was 8.6%, compared to 13.5% in the lowest Townsend quintile. Mosaic paints a worse picture of social disparities in smoking prevalence in the UK than previously-used measures of social class, and may be a useful tool for distinguishing the characteristics of groups with a particularly high smoking prevalence.

A Mosaic classification and/or Townsend quintile was missing for some patients in this analysis, though the odds of being a smoker for these are not extreme, suggesting that this data was missing at random and a high-prevalence group has not been missed. It is unclear why this information was missing for some people, but it may be that these patients' postcodes were not recorded by their general practice, and therefore EPIC was unable to map them to the area-based measures of deprivation.

An assumption has been made that patients not classified as current smokers are non-smokers, including those with no smoking-related Read codes documented in their medical records. This may lead to an underestimation of smoking prevalence. However, it has been shown that prevalence figures obtained using this assumption are reasonably reflective of those suggested by nationally-representative surveys[11], and the majority of patients with missing smoking records in THIN are either ex- or non-smokers[15]. The differences in the proportion of patients with a smoking record in different categories of each measure of deprivation were small and unlikely to have contributed to any great extent to the socio-economic differences in smoking prevalence reported.

The Mosaic classification, which groups individuals into 61 categories, may be quite a cumbersome system to use as a socioeconomic measure in most statistical models and, as noted already, little information is available from Experian about how the classification is derived, limiting assessment of the validity of their approach and the potential to replicate it. Some variables indicating individuals' health status, which may be related to past or current smoking behaviour, are used to derive Mosaic; this may confound identification of the groups most and least likely to smoke. In the interpretation of Mosaic it is also important to be aware that the classification is an area-level measure, based on postcode areas of approximately 15 households, and that the characteristics of any given type will only apply to the majority of individuals of that type - not all of them. It is also important to note that the estimated odds ratios presented in this study will overestimate the respective risk ratios across the groups as smoking prevalence is 20.8% overall, not a rare outcome. In calculating odds ratios, the use of the Mosaic group or type with the lowest smoking prevalence as the reference category will have maximised the difference in odds ratios observed across categories, though this is an appropriate approach for demonstrating the wider extremes in smoking prevalence that can be identified using Mosaic.

Those Mosaic types with a higher prevalence of smoking were characterised by minimal levels of education, low income, and manual occupations. These findings are consistent with existing knowledge, acquired using individual measures of socioeconomic status, such as income, education, and occupation[14]. However, Mosaic provides further detail - the Mosaic types with highest prevalence do not have access to a car, have little community support, are debt-ridden and tend to spend a lot of time in front of the television. Some of these factors seem likely to contribute to difficulty in quitting smoking, suggesting, perhaps, difficulty in accessing cessation support and advice. These findings provide some insights into how these groups might be targeted, such as through mobile smoking cessation services and provision of transport to enable access to existing services, or through television campaigns, utilising the principles and techniques of social marketing to ensure that smokers are targeted with appropriate cessation interventions[16]. There is some evidence that providing cessation services in novel settings, such as community pharmacies, dental surgeries or workplaces, may be effective in engaging large numbers of smokers, though more research is needed to determine whether these are successful in reaching disadvantaged groups in particular[17]. Similarly, mass media campaigns may have a valuable role to play in encouraging smoking cessation, though again there is limited evidence whether such campaigns are effective in reaching large numbers of the most disadvantaged smokers[18,19]. Given that many Mosaic groups with the highest smoking prevalence are in debt, offering financial incentives may provide a useful tool to engage these groups in cessation services. Existing research suggests such incentives may indeed increase the number of disadvantaged smokers who attempt to quit, and the number who succeed in doing so, though again further studies would be of benefit[17].

This study is one of the first to look at the association between Mosaic and smoking prevalence, and certainly the first to do so on such a large scale. In conclusion, the Mosaic classification system has been found to be a useful tool in examining the disparities in smoking prevalence between different socioeconomic groups within the UK, with those in the group with the highest smoking prevalence being over four times as likely to smoke as those in the group with the lowest prevalence. Mosaic is potentially useful for identifying the characteristics of groups of heavy smokers which can then be used to tailor cessation interventions to ensure these are as successful as possible and make the best use of resources. Though Mosaic only classifies individuals living in the UK, a similar approach to the use of market research and consumer segmentation intelligence may provide a means to identify groups of people with high smoking prevalence in other countries and target them with appropriate cessation interventions.

## Competing interests

The authors declare that they have no competing interests.

## Authors' contributions

SL and LS conceived the study, AS performed the statistical analyses and wrote the first draft of the manuscript, and all authors contributed to its critical revision and approved the final version.

## Pre-publication history

The pre-publication history for this paper can be accessed here:

http://www.biomedcentral.com/1471-2458/10/755/prepub
